# Centrioles and Ciliary Structures during Male Gametogenesis in Hexapoda: Discovery of New Models

**DOI:** 10.3390/cells9030744

**Published:** 2020-03-18

**Authors:** Maria Giovanna Riparbelli, Veronica Persico, Romano Dallai, Giuliano Callaini

**Affiliations:** 1 Department of Life Sciences, University of Siena, Via Aldo Moro 2, 53100 Siena, Italy; riparbelli@unisi.it (M.G.R.); persico@student.unisi.it (V.P.); romano.dallai@unisi.it (R.D.); 2 Department of Medical Biotechnologies, University of Siena, Via Aldo Moro 2, 53100 Siena, Italy

**Keywords:** Hexapoda, male gametogenesis, ciliary structures, centrioles

## Abstract

Centrioles are-widely conserved barrel-shaped organelles present in most organisms. They are indirectly involved in the organization of the cytoplasmic microtubules both in interphase and during the cell division by recruiting the molecules needed for microtubule nucleation. Moreover, the centrioles are required to assemble cilia and flagella by the direct elongation of their microtubule wall. Due to the importance of the cytoplasmic microtubules in several aspects of the cell life, any defect in centriole structure can lead to cell abnormalities that in humans may result in significant diseases. Many aspects of the centriole dynamics and function have been clarified in the last years, but little attention has been paid to the exceptions in centriole structure that occasionally appeared within the animal kingdom. Here, we focused our attention on non-canonical aspects of centriole architecture within the Hexapoda. The Hexapoda is one of the major animal groups and represents a good laboratory in which to examine the evolution and the organization of the centrioles. Although these findings represent obvious exceptions to the established rules of centriole organization, they may contribute to advance our understanding of the formation and the function of these organelles.

## 1. Introduction: The Centriole

Many aspects of a cell’s function depend on the dynamic behaviour of an organized microtubule network. Cytoplasmic microtubules are, indeed, involved in organelle motility and information delivery [1,2] and they represent a scaffolding structure needed for cell shape modification and cell polarity [3,4,5]. Moreover, the microtubules are actively involved in cell reproduction by building mitotic and meiotic spindles that support the proper cell division and guarantee the correct partition of the sister chromatids to the daughter cells [6]. Microtubule nucleation is ensured by g-tubulin [7,8] that may be present at different sites within the cell cytoplasm [9,10]. g-tubulin may be associated to Ran-GTP gradients around the chromatin [11,12,13,14], or recruited by augmin to pre-existing microtubules [15,16,17,18]. g-tubulin is also bound to the cytoplasmic face of the Golgi complexes [19,20], to distinct apical membrane domains in the developing *Drosophila* ommatidia [21] and of *Drosophila* tracheal cells [22]; and at hemidesmosomes of *C. elegans* epidermal cells [23]. However, the main site for microtubule nucleation in animal cells is the centrosome, a non-membrane bound organelle composed by several proteins, the pericentriolar material (PCM). The PCM is typically arranged in a higher-order structure made of fibres and matrices conserved from flies to humans [24,25,26].

The PCM undergoes a cell-cycle dependent expansion, a process termed centrosome maturation [27]. In this process, Spindle defective 2 (Spd2) and Centrosomin (Cnn), that are involved in the recruitment of g-tubulin, accumulated at the onset of cell division [28].

Thus, the increase of g-tubulin leads to an increase in the nucleation of both astral and spindle microtubules, which drive the assembly of a functional spindle.

At the heart of the centrosome there is a pair of centrioles, two microtubule-based barrel-shaped organelles of defined length and diameter [29], that warrants the integrity and the supramolecular organization of the centrosome itself. The coiled-coil proteins, pericentrin-like protein (PLP) and Cep152/Asterless form the scaffold for the matrix proteins Cep192/Spd-2, Cep215/Cnn and g-tubulin. It was observed PLP’s C termini are located close to the centriole wall [26].

In addition, to be the reference point for the organization of the centrosomal material, the centrioles may also act as templates for the axoneme assembly in cilia and flagella, that are involved in signalling and motility [30]. Therefore, the proper organization and dynamics of the centrioles are mandatory to ensure healthy cell life. Structural anomalies of the centrioles are found in several human cancers [31,32,33,34,35,36] and can be the cause of a spectrum of pathologies spanning from infertility to ciliopathies [37,38].

Since, the centrioles impact upon several aspects of cell development and physiology, their structure and function have been studied over the years. However, this analysis was mainly addressed to examine centrioles in a few model organisms, such as *Chlamydomonas reinhardtii*, *Caenorhabditis elegans*, *Drosophila melanogaster*, and some vertebrate cell lines. From these studies emerge a highly conserved organization of the centrioles. However, investigations in different animal groups revealed distinct and sometimes important differences in the architecture of the centrioles [39,40]. Therefore, the analysis of the variations occurring to the general structural plane of the centrioles could provide a unique opportunity to better understand their function and their assembly process. To gain insight into the general organization of the centrioles we review the centriole structure and function in Hexapoda, with attention to centriole dynamics during male gametogenesis. Hexapoda, among animal groups, exhibits greatly diversified spermatozoa. This is likely due to the old origin of the Hexapoda [41,42] and to their short lifespan that allows the accumulation of mutations and leads to faster genetic divergence [43]. Moreover, the genes involved in sexual reproduction tend to diverge faster than those codifying for components of non-reproductive tissues [44]. This leads to the rapid evolution of fertilizing barriers between different populations, with a consequent rapid sperm diversification [45].

## 2. Centriole Dynamics in Insect Spermatogenesis: Rule or Exception?

Centriole duplication must be accurately monitored during the cell cycle to avoid additional centrioles and, therefore, too many centrosomes that may result in multipolar spindles. Multipolar spindles are dangerous to tissue development and homeostasis, since they can lead to apoptosis or chromosomal instability, a hallmark of tumorigenesis [36,46,47,48,49]. Thus, the centrosome number is tightly regulated in somatic cells and the centrioles duplicate only once during the cell cycle [36]. The duplication of the centrioles is a semiconservative process that mirrors the DNA replication time. However, whereas DNA replication is restricted to the S-phase, the duplication of the centrioles usually starts at the G1/S transition and ends in G2 [50]. The case of Hymenopteran males, in which the haploid somatic cells have the usual centriole number, suggests that ploidy and centriole duplication could be unrelated.

Male gametogenesis in most of organisms consists of two consecutive cell divisions giving origin to four spermatids that differentiate in mature sperm. Since the spermatids have a haploid chromosome complement, the DNA replication must occur only one time at the beginning of the meiotic process. Surprisingly, the centriole cycle is uncoupled from the DNA replication and the centrioles duplicate during the second meiotic division in the absence of DNA synthesis. Thus, each haploid spermatid inherits a centriole pair as it usually occurs during the mitotic divisions in which the centriole cycle is tightly coupled to the DNA cycle.

**Figure 1:**
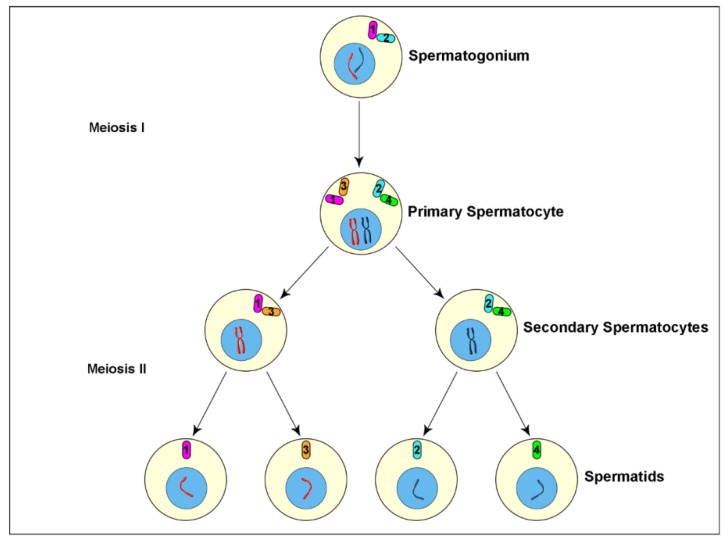
Cartoon of insect spermatogenesis depicting centriole and chromosome inheritance during the meiotic divisions. The four spermatids derived from each primary spermatocyte obtain centrioles that differ among them for their age and their parents. (1) Mother centriole; (2) Daughter centriole; (3) New daughter from the old mother; (4) New daughter from the old daughter.

The male meiosis in insects does not differ substantially from male meiosis of other organisms, but the centriole replication cycle represents a noticeable exception. Spermatogenesis usually starts with the asymmetric division of the germline stem cells at the apex of the testis. One daughter cell maintains stemness whereas the sister cell undergoes a variable number of spermatogonial mitoses leading to the formation of primary spermatocytes. The primary spermatocytes enter meiosis and form haploid spermatids that undergo a differentiation program to give origin to mature sperm. The centrioles do not duplicate during the second meiosis resulting in spindles that contain only one centriole at each spindle pole [40,51,52,53,54,55,56]. Consequently, the spermatids will receive at the end of the meiosis only one centriole that will be the basal body for the sperm axoneme. Remarkably, there is an age-related difference between the centrioles of the four spermatids that originate from each primary spermatocyte. Each primary spermatocyte inherits at the end of the spermatogonial mitoses a pair of centrioles, the mother and the daughter. These centrioles duplicate in early prophase and each parent becomes a mother for a new daughter. At the end of the first meiosis, one secondary spermatocyte inherits a pair of centrioles with the older mother, whereas the other spermatocyte receives a centriole pair with the older daughter. So, the spermatids obtain different aged centrioles (Figure 1). It is unclear if the intrinsic centriole asymmetry could have some impact on the fate of the mature sperm. During mitosis, also, the sister cells inherit unequal centrosomes with different aged centrioles [57]. The unusual centriole cycle observed in insect male gametogenesis suggests the presence of a surveillance mechanism during the second meiosis. This mechanism could prevent centriole duplication in the absence of DNA replication, thus ensuring the inheritance of a single centriole by the differentiating spermatids.

**Figure 2:**
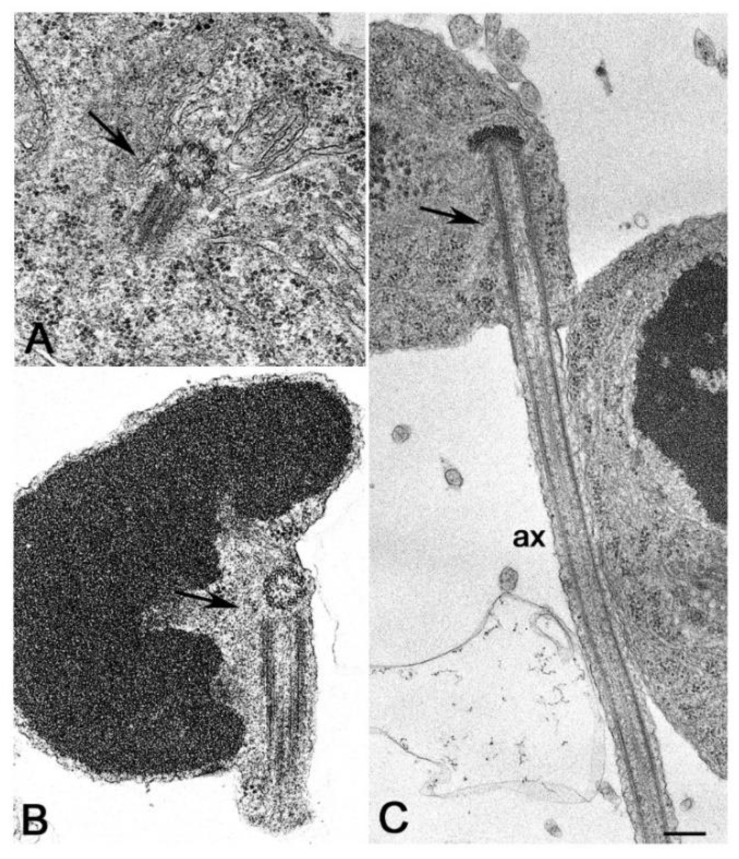
Abnormal male gametogenesis in the springtail *Allacma*. (**A**) Detail of one of the two centriole pairs in young primary spermatocytes. (**B**) Degenerating germ cell with two centrioles. (**C**) Spermatid with one centriole nucleating a functional axoneme (ax). Arrows point to centrioles. Bar: 200 nm.

However, every rule has its exceptions. There are, indeed, a few cases of insect spermatids that inherit a centriole pair. This condition follows an aberrant meiotic process that warrants the development of haploid species [58]. The spermatogenesis in the honeybee and the springtail *Allacma* also consists of an abortive first meiotic division, followed by an unequal division of the secondary spermatocyte. However, the spermatids inherit only one centriole in these species. The centrioles in the male germ cells of the honeybee seem to replicate twice during the first meiosis and as a result, each primary spermatocyte contains sixteen centrioles. However, the supernumerary centrioles are eliminated through cytoplasmic blebs prior to the second meiotic division and the spermatids contain only one centriole [59,60]. The primary spermatocytes of *Allacma* have the usual number of four centrioles (Figure 2A) that give rise to two daughter cells, each with a centriole pair (Figure 2B). However, one of the sister cells does not divide and soon degenerates maintaining the centriole pair, whereas the other undergoes a normal division. Thus, each spermatid daughters inherits one centriole that will assemble a functional sperm axoneme (Figure 2C) [61].

## 3. The Case of *Drosophila:* Three Centriole Models?

Many studies deal with the structural and the molecular organization of the centrioles in various *Drosophila* tissues [62,63], making this organism one of the best suitable models for centriole/centrosome research. Although, the *Drosophila* centrioles assemble following a conserved molecular program, they slightly differ from the centrioles of vertebrate tissues. The mother centriole in vertebrate cells has distinct appendages [64]. The distal appendages are involved in membrane docking during primary cilia assembly [65] whereas the subdistal appendages represent the focus for the recruitment of the pericentriolar material required for microtubule nucleation [66]. A distinct cartwheel is transiently present in the basal region of the daughter centrioles. In contrast, the parent centrioles of *Drosophila* do not display apparent morphological differences [67] and maintain a distinct cartwheel, apart from the ciliated sensory neuron centrioles [68]. Therefore, the parent centrioles can be only recognized by their relative spatial disposition, with the daughter orthogonal to the proximal end of the mother [69]. However, the mother and daughter centrioles in *Drosophila* may be endowed with different functional aspects and their different age impacts with the activity of the centrosome and has important consequences on the binary decision fate of the sister cells [70,71,72,73].

Despite the parent centrioles in *Drosophila* tissues are morphologically undistinguishable, a subtle molecular asymmetry has been occasionally reported. For example, the centrosomal protein PLP accumulates preferentially to the mother centrioles of somatic cells [74,75], whereas centrobin is recruited by the daughter centrioles in larval neuroblasts [76], sensory neurons [77] and ommatidial cells [21].

It has been reported that centrobin is associated to the daughter centrioles in female germ stem cells [78] and during the spermatogonial divisions, but this protein is found at the basal region of both the parent centrioles in primary spermatocytes [79]. This observation that points to the loss of parental identity among the centrioles is supported by the finding that both the parents are able to organize cilium-like structures and sperm axonemes [80,81]. These functions in vertebrate cells are reserved to the mother centriole. A transient enrichment of the centriole duplication factor Ana2 has been observed to the daughter centrioles in primary spermatocytes [82]. Moreover, the spindle assembly abnormal 4 (Sas4) and the spindle assembly abnormal 6 (Sas6), two conserved proteins mainly involved in centriole assembly and elongation, display an asymmetric accumulation to the proximal end of the parent centrioles. Remarkably, these proteins are enriched at the basal regions of the daughter centrioles during male gametogenesis, whereas they accumulate to the mother centrioles in somatic tissues [83], supporting a subtle molecular diversity among germline and somatic cell centrioles.

**Figure 3:**
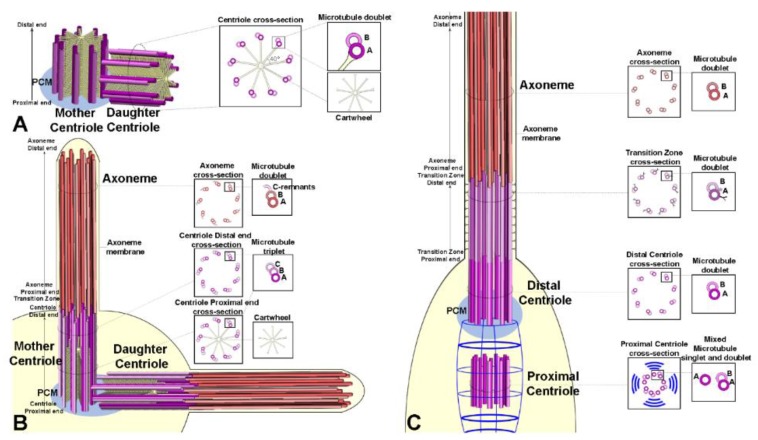
Schematic representations of the centriole architecture in *Drosophila* somatic cells (**A**), primary spermatocytes (**B**) and sensory neurones (**C**). Ciliary projections of both spermatocytes and sensory neurons are also depicted. (**A**) The somatic centrosome of *Drosophila*, such as vertebrate centrosome, consists of two centrioles, one mother and one daughter, arranged orthogonally to each other, and the pericentriolar material around them (PCM, light blue). Even then both centrioles show a proximal-distal organization, the centriole wall is composed of nine microtubule doublets, each of which presents the complete tubule A, and the tubule B. (**B**) In *Drosophila* male gametogenesis, mother and daughter centrioles act both as basal bodies for the ciliary projections. Both the centrioles are composed of nine microtubule triplets and have a distinct cartwheel in their proximal region. The axoneme of the ciliary projections is composed of nine microtubule doublets and the C-remnants. The cell cytoplasm is represented in light yellow. PCM, pericentriolar material in light blue. (**C**) In *Drosophila* sensory neurons, the distal and the proximal centrioles are coaxial and lack a distinct cartwheel. The ciliary rootlet (in blue) emerges from the proximal end of the distal centriole and form a cage enclosing the proximal centriole. The proximal centriole consists of mixed singlet and doublet microtubules and it is smaller than the distal centriole that is built by doublet microtubules. The DBB forms an elongated transition zone (TZ) characterized by short lateral projections (in grey) and Y-like structures. The axoneme nucleated by the distal centriole is composed of nine microtubule doublets. The cell cytoplasm is represented in light yellow. PCM, pericentriolar material in light blue.

Centrioles in embryos [67], somatic tissues [84], and cultured cells [85] are very short cylinders (about 0.2 mm in length and diameter) and consist of nine doublet microtubules with a distinct central cartwheel (Figure 3A). Mother and daughter centrioles are orthogonal, and they disorient at the onset of anaphase before entering the duplication process [21,86].

The centrioles of the male and female germ cells invariably consist of nine triplet microtubules [84,87]. These centrioles are very short (about 0.2 µm) during the asymmetric division of the germline stem cells and the spermatogonial mitoses but reach the length of about 1 µm at the end of the first prophase (Figure 3B).

The pole cells, precursors of the germ line stem cells, have centrioles with nine doublet microtubules [88], whereas the centrioles of the gonioblasts have triplets. We could expect that the transition from doublets to triplets might occur within the stem cell niche. Interestingly, the old mother centriole, localized to the apical cytoplasm of the male germline stem cells, is composed of triplets, whereas its daughter always consists of mixed doublets and triplets [84].

What it is the meaning of the different microtubule numbers making the centriole wall of somatic and germ cells? One possibility is that the centrioles with triplet microtubules are correlated to the presence of ciliary structures that at the onset of spermatid differentiation elongate in the sperm axoneme [80,89]. This could be the case of male germ cells. However, the female germ cells have centrioles with triplet microtubules [87], albeit they lack ciliary projections. Moreover, the ciliary axoneme of auditory and olfactory sensilla are assembled from basal bodies composed of doublet microtubules. However, the growth of centrioles and axonemes is severely affected in *uncoordinated* (*unc*) *Drosophila* mutant spermatocytes in which the C-tubule dynamic is defective [80]. Thus, the C-tubule seems to play a functional role only during male gametogenesis.

Unlike centrioles of germ cells and somatic tissues, the centrioles of auditory and olfactory sensilla display a distinct dimorphism (Figure 3C). The parent centrioles were unusually aligned in tandem and easily recognizable [90,91]. The distal centriole that nucleates the ciliary axoneme is built by nine doublet microtubules [92], whereas the proximal one is composed of mixed singlets and doublets [77]. This suggests that the proximal centriole does not complete its wall during the development of the sensory organ. Moreover, the distal centriole is longer than the proximal centriole which is enveloped by fibrous rootlets emerging from the proximal end of the distal parent. These centrioles represent a noticeable exception to the general architecture of the centrioles in *Drosophila*. The parent centrioles of somatic and germ cells display a distinct cartwheel that persists along their life, whereas the tandem aligned centrioles of the sensory organs lack this structure. It is possible that the cartwheel loss may be part of the complex differentiation program of the sensory neurons. Centrioles consisting of mixed doublets and triplets, but lacking a distinct cartwheel are found in the post-mitotic cells that form the hub region of the male stem cell niche [84]. The cartwheel loss may represent an intermediate stages of centriole degeneration. Accordingly, centriole elimination in the *Drosophila* eye begins with the gradual disassembly of the microtubule wall, followed by the disappearance of the cartwheel and the loss of the nine-fold symmetry [21].

## 4. Centrioles in Hexapoda: Common and Uncommon Models

**Figure 4:**
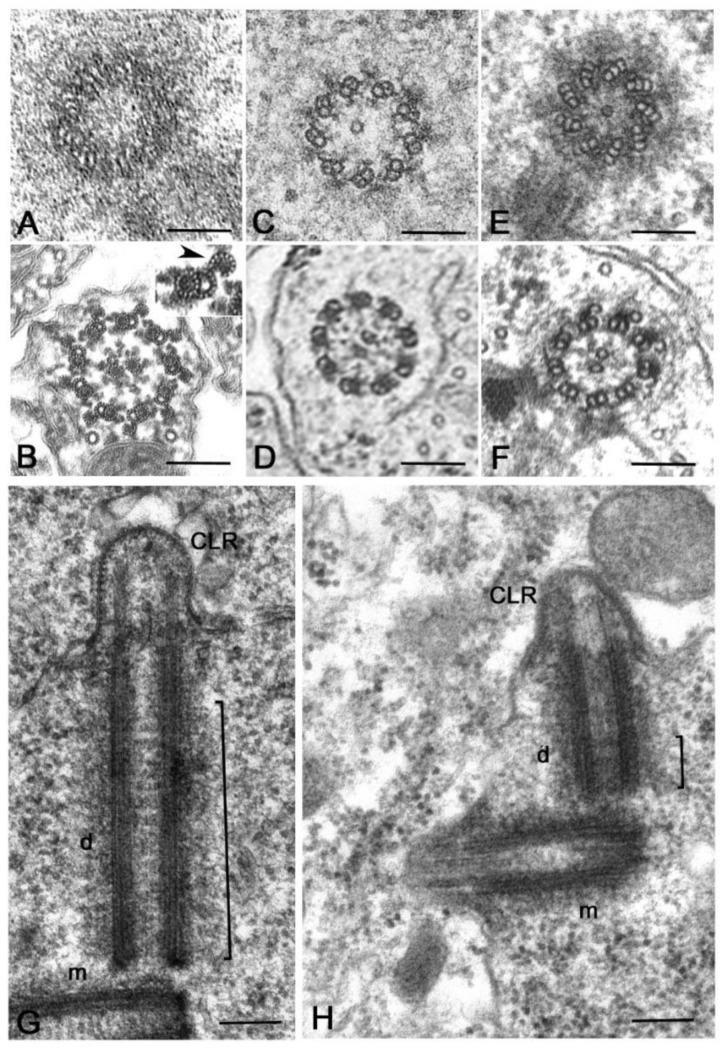
Common centriole models in germ cells of Hexapoda. Cross sections of centrioles and axonemes in (**A**,**B**) *Campodea* (Diplura), (**C**,**D**) *Allacma* (Collembola), and (**E**,**F**) *Drosophila* (Diptera). Low Hexapoda have centrioles with doublet microtubules (**C**) and axonemes without accessory tubules (**D**). Insect spermatocytes usually show centrioles with triplet microtubules (**A**,**E**) and axonemes with distinct accessory tubules (B,F) derived by the proliferation of the C-tubules (inset B, arrowhead). Longitudinal sections of centrioles in growing primary spermatocytes of the coleopteran *Adalia* (**G**) and the dipteran *Drosophila* (**H**) showing their different length: both the centrioles and the cilium-like regions (CLR) will grow further during prophase progression to reach their full length in mature primary spermatocytes but their different dimensions do not change. The cartwheel (brackets) extends along the length of the centriole in *Adalia*, whereas it is restricted to the proximal region of the centriole in *Drosophila*. The daughter centriole (d) is orthogonal to the basal region of the mother (m). Bars: A–F, 100 nm; G,H, 200 nm.

Regardless of the careful analysis of centriole biogenesis and structure in *Drosophila* only a few studies deal with the organization of the centrioles in other Hexapoda. Most of the reports are focused on the sperm structure, but the centriole undergoes important modifications during axoneme elongation, and its former architectural plane may not be longer visible in mature sperm. It has been reported that the spermatid centriole of some insect species has a conventional pattern of nine triplets [92], whereas the sperm basal body in these species is composed by nine doublets [93]. An axoneme consisting of nine peripheral doublets, two central microtubules and nine accessory tubules (9 + 9 × 2 + 2) is the prevalent model within the insect sperm and represent an important synapomorphy among the group [58]. The centriole templates the axoneme architecture by the elongation of its A- and B-tubules and the peripheral accessory tubules arise as modifications of the C-tubule [94] (Figure 4A,B). Thus, it is conceivable that these centrioles initially consist of nine triplet microtubules.

The basal hexapods Collembola have centrioles with nine doublet microtubules and their sperm axoneme lacks accessory tubules (Figure 4C,D) [61]. True accessory tubules forming an outer ring around the axoneme are firstly observed in the basal group Diplura that have centrioles consisting of nine triplet microtubules [95] and then in higher insects (Figure 4E,F). The accessory tubules, when present, can display a variable diameter due to their different protofilament number that ranges from 13 to 40 [58]. Therefore, the basal Hexapoda have centrioles with doublet microtubules, whereas centrioles with nine triplet microtubules represent the more diffuse model within the insects, even if some exceptions have been occasionally observed.

Elongated centrioles with nine-fold symmetry have been reported in some coleopterans [96] and neuropterans [97]. Remarkably, these centrioles have a very extended cartwheel (Figure 4G). In *Drosophila*, the germ stem cells and spermatogonia have small centrioles in which the cartwheel extends along their length. However, the cartwheel is restricted to the proximal end of the centrioles when they grow during the first prophase to increase tenfold their length (Figure 4H). This points to the presence of a mechanism monitoring the elongation of the cartwheel during male meiosis and suggests that this control could be overcome in some insect species that display very elongated cartwheels. A very strange condition has been recently reported in larval tissues of the male wasp *Anisopteromalus* in which cylindrical structures with nine-fold symmetry but without microtubules would be present [98]. Surprisingly, adult tissues of this species display usual centrioles with triplet microtubules and nine-fold symmetry.

**Figure 5:**
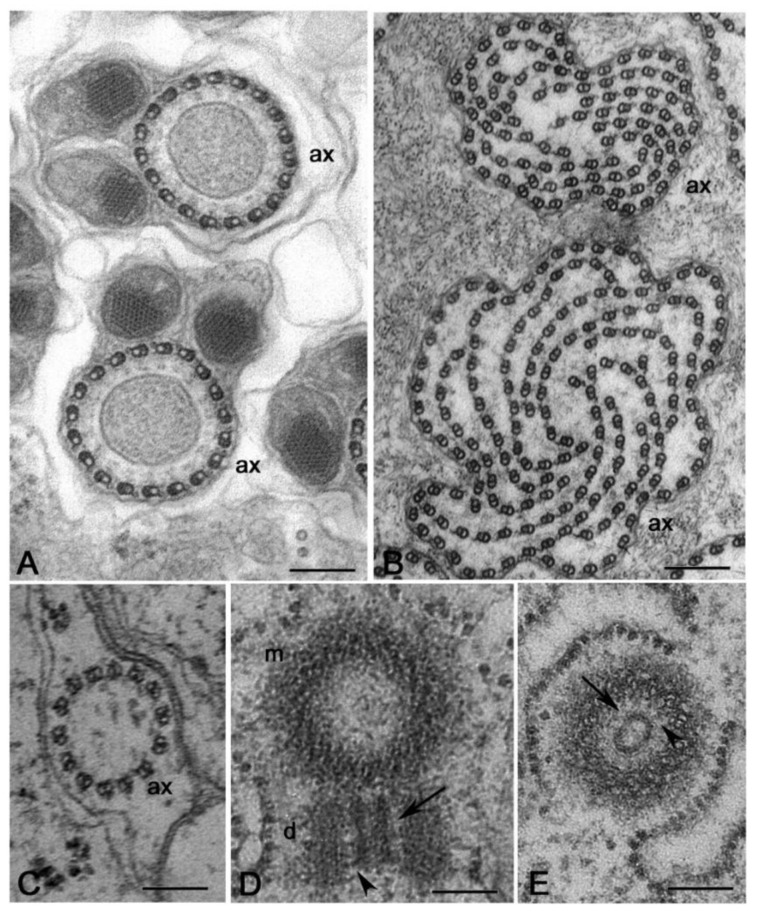
Axonemes in Hexapoda diverging from the conventional 9+2 model. (**A**) Cross sections of the axonemes of the spermatids in the cecidomyiids *Anaretella* showing a circular array of doublets and (**B**) *Monarthropalpus* with many doublets arranged in concentric rows. (**C**) Spermatid of the proturan *Acerentomon* with an axoneme consisting of 14 doublet microtubules. (**D**,**E**) Cross sections of centrioles in the primary spermatocyte of *Acerentomon* showing that their wall consists of 14 doublet microtubules. The daughter centrioles have an inner large tubule (arrows) connected to the peripheral doublets by thin links (arrowheads). ax, axoneme; d, daughter centriole; m, mother centriole. Bars: 200 nm.

It is hard to imagine the structural organisation of the spermatocyte centrioles in some dipteran groups, like Cecidomyiidae that have large flagellar axonemes consisting of a highly variable number of microtubule doublets (Figure 5A,B) [99]. It is unclear whether these aberrant axonemes reflect the structure of unusual centrioles or if they are the result of significant modifications of the centriole architecture occurring during the initial stages of spermatid differentiation. The sperm axoneme of the gall-midge *Asphondylia* consists of a bundle of about 2000 doublet microtubules that originate from sparse clusters of electron-dense material associated with elongated cisternae [100], but residual centrioles have not been detected in the elongating spermatids. There is, therefore, a compelling need to re-examine the formation of these aberrant axonemes to understand the structure of the centrioles present during early stages of gametogenesis in these insects. This analysis could uncover new models of centriole geometry, useful to reveal highly conserved mechanisms managing centriole biogenesis and evolution.

Ultrastructural analysis of the male gametogenesis in *Sciara*, that also displays a giant axoneme consisting of a spiral of about 70 doublet microtubules, reveals the presence in primary and secondary spermatocytes of extraordinarily large centrioles consisting up to 70 doublet microtubules [101]. A similar condition has been described during the spermatogenesis of the proturan *Acerentomon,* that displays unusual axonemes (Figure 5C) and centrioles (Figure 5D,E) with 14 doublet microtubules [102]. However, whereas the centrioles in *Acerentomon* have a central hub-like structure, the giant centrioles of the *Sciara* spermatocytes do not have a distinct hub, but, in its place, they show a large cylindrical sheet from which emerge thin fibres that contact the peripheral doublets. The presence of such large centrioles raises the question of how the symmetry could be achieved and maintained. Typical centrioles have, indeed, a high evolutionary conserved nine-fold symmetry ensured by a cartwheel consisting of a central hub and nine radial spokes. The regular organization of the cartwheel is due to Sas6, a protein that consists of three domains: an amino-terminal globular head domain, a coiled-coil region and a carboxy-terminal region. The coiled-coil region determines the dimerization of Sas6 monomers into homodimers that form the radial spokes, whereas the head domain mediates the interaction between Sas6 homodimers forming the central hub. This arrangement leads to the assembly of Sas6 into a nine-fold symmetric cartwheel [103,104,105,106,107,108].

Remarkably, the fibres emerging from the large hub of *Acerentomon* and the cylindrical sheet of *Sciara*, are similar in length to the radial spokes found in *Drosophila* somatic and germ cell centrioles that have a conventional nine-fold symmetry [102]. Therefore, the giant centrioles found in *Sciara* and *Acerentomon* spermatocytes may reflect the presence of a Sas6 protein with different properties. Sas6 homodimers with angles larger than those found in canonical centrioles could be assembled and then larger hub-like regions could be formed. The coiled-coil tail of the Sas6 protein could be conserved to assemble radial spokes of constant length in both canonical and giant centrioles. Interestingly, only the daughter centrioles display the hub-like structure in *Acerentomon* (Figure 5D,E). The daughter centrioles with 14 doublet microtubules are disposed in spermatogonial cells at right angles to their mothers that have nine doublet microtubules. Thus, their diameter is greater than the length of their mothers [102]. The finding that centrioles with nine-fold symmetry are mothers for giant ones rules out a “template” process for centriole duplication and calls into question the recent hypothesis that the cartwheel assembled within the lumen of the mother centriole could represent the building block for the formation of a new daughter [109].

The observation that centriole geometry varies not only from different species and among various tissues of the same organism but also within the same cell, raises important questions on maintenance and the propagation of the 9-fold symmetry through evolution and its central role in the conserved functions of the centrioles.

## 5. Centriole Overduplication in Insects: Breaking the Rules

The organization of a functional mitotic spindle in proliferating cells requires the presence of two centrosomes that originate by the duplication of the single centrosome inherit at the end of the previous division. Since centrosome assembly and maintenance depend on the centriole pair present inside, the behaviour of the centrosome is closely related to the biogenesis of its centrioles. The duplication of these organelles usually starts before DNA duplication at the transition between G1 and S phases when a new procentriole grows perpendicularly from a single site at the proximal end of each mother [50,110]. Mandatory for the duplication process is the loss of the orthogonal orientation of the parent centrioles, the disengagement, that starts at metaphase/anaphase transition of the previous cell cycle. This physical separation is generally retained to be the licensing factor that enables the reproduction of the centrioles at the following interphase and warrants their correct number during the cell cycle, thus avoiding unwanted duplications [111]. It has been shown, indeed, that centrioles become competent to duplicate only when they are separated [112].

**Figure 6:**
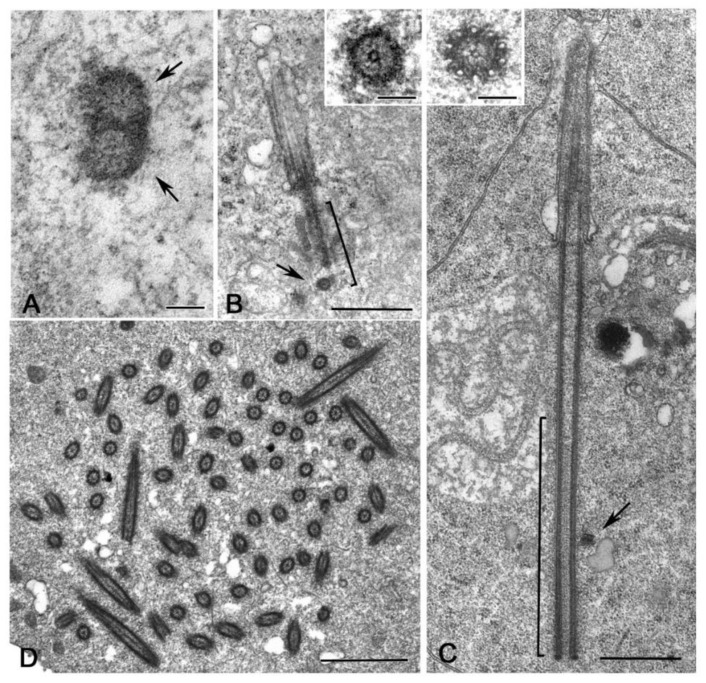
Abnormal centriole duplication in insect male germ cells. (**A**) Detail of two procentrioles (arrows) in the primary spermatocytes of the butterfly *Pieris*. Procentrioles (arrows and insets) in young spermatids of the fruit fly *Drosophila* (**B**) and the ladybird *Adalia* (**C**) brackets outline the extension of the centrioles. (**D**) Multiple centrioles in the spermatids of the termite *Mastotermes*. Bars: A, insets B and C, 100 nm; B,C, 500 nm; D, 1 mm.

It is surprising, therefore, the finding of distinct procentrioles close to the basal region of each parent centriole in primary spermatocytes of the lepidopterans *Bombyx* [113], *Ephestia* [114] and *Pieris* [115] that have orthogonal or V-shaped engaged parent centrioles. Procentrioles in *Pieris* look like early intermediates in that they consist of a central cartwheel and a ring of peripheral electron-dense material in which an incomplete number of singlet microtubules has been observed (Figure 6A) [115]. They do not behave like true procentrioles, but their development halts at an early stage before the assembly of a complete A-tubule set. Distinct clusters of up to four procentrioles were often seen in the proximity of a single parent centriole in butterfly spermatocytes. This is in contrast to the traditional model in which the assembly of the daughter centriole is spatially restricted to a defined site close its mother to warrant a centriole copy number control [116,117]. Therefore, the centrioles of the primary prophase spermatocytes undergo an extra duplication cycle, in addition to their usual replication that occurs in early prophase. The observation of multiple procentrioles at each spindle pole in butterflies suggests that the rule of ‘only one centriole per mother’ is downregulated and that the assembly of a daughter centriole does not necessarily prevent the formation of additional ones. This finding also questions the proposed model in which the cartwheel of the procentriole acts to prevent the reduplication of the parent [118]. Presumably, the supernumerary procentrioles play a redundant role, since their structure is unmodified during meiosis and they disappear on spermiogenesis. Intriguingly, thin fibrous material links the basal regions of just duplicated parental centrioles in butterfly and *Drosophila* young primary spermatocytes [94]. This material persists in mature spermatocytes when the centrioles have reached their full length [119]. Inhibition of Polo/Plk1 in early *Drosophila* spermatocytes by the dihydropteridinone derivative BI2536, an ATP-competitive kinase inhibitor, leads to the failed separation of the parent centrioles. This suggests that Polo kinase regulates the proteolysis of the linkage among the parent centrioles to ensure their disengagement [120]. Centriole duplication without disorientation has been also shown in mammalian cultured cells expressing high levels of active PLK1 [121]. This suggests that centriole duplication without disengagement is not a paradox restricted to some insect spermatocytes, but it is also present in mammalian cells under certain conditions.

An unexpected centriole duplication is also observed in early spermatids of some insect species in which a structurally distinct procentriole is observed close to the proximal region of the basal body at the beginning of the axoneme elongation (Figure 6B,C) [96,122,123]. The assembly of these procentrioles requires in *Drosophila* typical centriolar proteins, such as Ana1, Bld10/Cep135, Sak/Plk4 and Sas6 [82,124,125]. However, the procentrioles do not elongate and do not acquire B and C tubules. The variable position of the procentrioles along the length of the basal body points against the presence of a fixed site of daughter centriole assembly close to the proximal end of the mothers. The observation of procentrioles in Diptera, Coleoptera and Lepidoptera suggests that these structures may be largely diffused during male gametogenesis of insects. However, further studies are required to verify this possibility.

An extreme condition has been observed during the male gametogenesis of the termite *Mastotermes* in which the spermatid inherit one centriole, but soon during early spermiogenesis about hundred centrioles are assembled (Figure 6D) [126,127]. Apart gametes of lower plants [128,129], multiple centrioles in the spermatids have until now reported only in gastropods [130] and annelids [131]. It is unclear how the dramatic increase in centriole number occurs in *Mastotermes* spermatids. The short time needed to achieve one hundred centrioles points to a process of assembly like that found during basal body formation in multiciliated cells. However, clusters of dense material resembling deuterosomes, the transient structures need to the massive assembly of centrioles in multiciliated cells [132,133,134], have never observed in *Mastotermes*. Rather, scattered isolated centrioles at different stages of elongation are found. This suggests that a process of *de novo* assembly like that observed in parthenogenetic eggs could operate in *Mastotermes* spermatids. However, it has recently been shown show that deuterosomes are dispensable for centriole amplification during multiciliogenesis of some mouse and *Xenopus* cell types where a high number of procentrioles is formed in the vicinity of the parent centrioles [135]. Since orthogonally oriented centrioles are also found in *Mastotermes* spermatids it is possible that a such mechanism of rapid duplication could operate in this system to enable the assembly of many centrioles without the presence of deuterosomes.

## 6. Unusual Ciliary Structures in Hexapoda Spermatocytes

Insects lack conventional primary cilia but have ciliary structures within the sensory neurons [136,137] and ephemeral cilium-like projections associated with developing epidermal secretory cells [138,139]. It has been observed that in *Drosophila* the ciliary projections of neurons of type I elongate through a mechanism mediated by an intraflagellar transport (IFT) mechanism [140,141], namely the compartmentalized pathway of assembly [142]. The base of the sensory cilia in *Drosophila* displays Y-links [68,90] and contains some conserved transition zone (TZ) module proteins [89,90,91,143]. This suggests that this region can be regarded as a typical TZ, a specialized domain found at the boundary between the basal body and the axoneme in primary cilia and directly involved in their assembly and maintenance.

**Figure 7:**
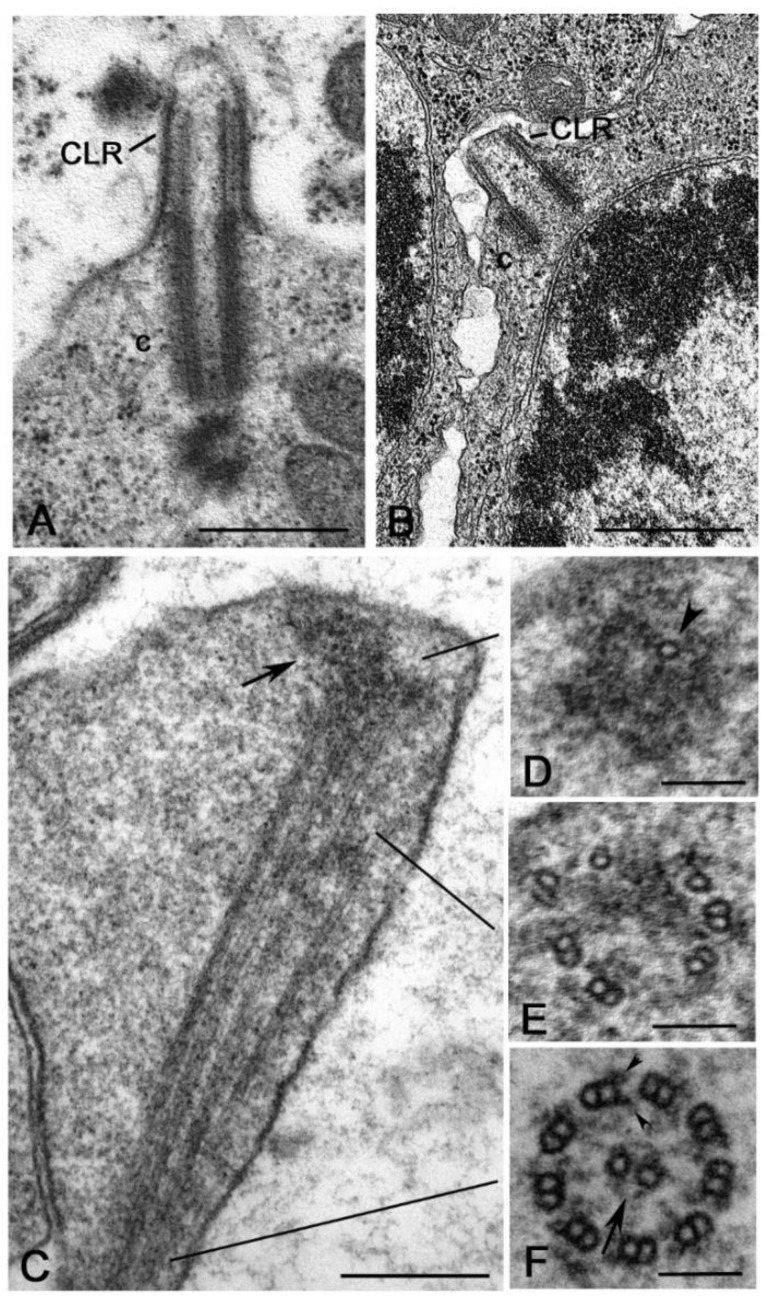
Ciliary projections in the male germ cells of Hexapoda. (**A**) Mature *Drosophila* primary spermatocytes showing full length centrioles (c) projecting out the plasma membrane to assemble a distinct cilium-like region (CLR). (**B**) Primary spermatocyte of *Acerentomon* with a short centriole (**C**) and CLR. (**C**) Longitudinal section of the distal enlarged region of a ciliary projection in the butterfly *Pieris*: note the apical cluster of electron-dense material (arrow) in which the tips of some microtubules end. (**D**,**E**,**F**) Cross sections of the ciliary projections showing the gradual organization of the axoneme from the apex to the basal region. (**D**) Distal sections show a few single tubules (arrowhead) within the electron-dense material. (E) Sections at a lower level display complete and incomplete doublet of microtubules. (**F**) Proximal sections show complete axonemes consisting of nine doublets of microtubules with dynein arms (arrowheads) and the central tubules (arrow). Bars: A,B, 500 nm; C, 250 nm; D–F, 100 nm.

In addition to the sensory cilia, *Drosophila* has another type of ciliary structures, the so-called cilium-like regions (CLR) observed during male gametogenesis (Figure 7A). After the four spermatogonial divisions, each primary spermatocyte inherits a centrosome that soon duplicates. So, at the beginning of the first meiotic prophase, the germ cells have two pairs of short centrioles [81]. Early in prophase, the parent centrioles migrate toward the cell surface where each of them organizes the axoneme of a CLR that protrudes from the plasma membrane [144,145]. The centriole/CLR complexes increase in length during prophase and persist throughout meiosis to organize the meiotic spindle [80]. It is still unclear how centrioles can elongate whereas they are engaged in the assembly of the axoneme. At the end of meiosis, the centriole/CLR complexes are inherited by the spermatids and will be the precursor of the sperm flagellum [80].

Although a detailed analysis of the centriole behaviour has not been reported during the spermatogenesis of other insects, the presence of CLRs has occasionally been observed within different Hexapoda groups. The CLRs share a common axoneme scaffold, but their size and dimension could vary among different species. The large centrioles of the primary spermatocytes of the proturan *Acerentomon* extend their distal region to push against the cell membrane and form short CLRs (Figure 7B) [102]. CLRs like those observed in *Drosophila* spermatocytes have been described during the spermatogenesis of some coleopterans [95] and neuropterans [97]. Interestingly, the primary spermatocytes of the crane fly *Nephrotoma* (Diptera) [146], the caddisfly *Potamophylax* (Tricoptera) [147] and some lepidopterans, such as *Bombyx* [113,148], *Ephestia* [114] and *Pieris* [115], have extraordinarily elongated CLRs. These structures have been also observed at the beginning of the last century [149,150] and reviewed in [151].

The dynamics of the CLRs in insect spermatocytes break from the general rule of the primary cilium biology observed in vertebrate cells, where this organelle has a transient life correlated to cell cycle progression and its presence is incompatible with cell division [48,152,153]. On the contrary, in insect spermatocytes, the CLRs do not disassemble at the beginning of the meiotic divisions, but they are internalized, and the centrioles organize the centrosome that drives the assembly of the spindle poles. Moreover, all the parent centrioles assemble ciliary projections in insect spermatocytes, whereas in vertebrate cells only the mother centriole can nucleate a ciliary axoneme and the daughter acquires this ability during the next cell cycle.

An additional question is the elongation mechanism of the CLRs. Although the CLRs of *Drosophila* spermatocytes diverge from the conventional primary cilia, they share with vertebrate cilia some conserved TZ module proteins [87,88,89,143,154]. The CLRs are assembled in *Drosophila* by IFT-independent mechanisms [155] and it has been proposed that their grow requires components directly recruited from the cytoplasm, namely the cytosolic pathway of assembly [142]. However, this diffusion mechanism may be working when the CLRs are relatively short, as in the case of *Drosophila*, but it could be inappropriate when the CLRs are very elongated as those observed in the spermatocytes of crane flies, caddisflies and several lepidopteran species. Presumably, alternative processes are required to enable the assembly of these extremely long axonemes. The initial stages of axoneme formation in lepidopterans mirror the process described in *Drosophila* in which the A- and B-tubules of the centrioles elongate and push against the plasma membrane. However, while the diameter of the CLR is roughly constant in *Drosophila* spermatocytes, the elongated CLRs of lepidopteran spermatocytes display a distal dilation [156,157]. This distal swelling contains an electrodense material in which some scattered microtubules are immersed (Figure 7C). Moving from the tip to the basal region of the ciliary projection, the microtubules are seen as singlets (Figure 7D), then they are arranged in distinct doublets (Figure 7E) and finally they organize a complete axoneme (Figure 7F) [157]. This suggests that the microtubule plus ends could grow within the dense material at the distal end of the CLR. The CLRs of lepidopterans modify dramatically its structure during meiotic progression and acquire a central tubule pair and dynein arms [114], thus resembling the axoneme of an elongating spermatid. The presence of dynein arms points to the active beating of this structure. But if so, why a primary spermatocyte should have a motile axoneme? Distinct Y-links have been observed at the transition between the centriole and the ciliary region in *Ephestia*. It could be important to observe that similar Y-links are found in *Drosophila* sensory neurons in the association with an IFT-dependent mechanism of axoneme assembly. However, the presence of IFT trains during the assembly of the axoneme in *Ephestia* is unclear.

## 7. Exceptions to the Conventional Presence of Sperm with Single Centrioles

Centriole duplication during the second meiosis leads, in most of the organisms, to the formation of haploid spermatids with a centriole pair, but, as a rule, only one centriole, the mother one, organizes the sperm axoneme [45,56]. It is unclear why the daughter centriole is unable to nucleate an axoneme. Perhaps, the parent centrioles maintain during spermatid differentiation an intrinsic asymmetry responsible for different functional properties. Remarkably, the spermiogenesis in mammals circumvents this general rule and the sperm axoneme is organized by the daughter centriole [158], that is dramatically remodelled in structure and composition during sperm maturation [159].

On the contrary, at the end of meiosis, most of the insect spermatids inherit only one centriole, that could be the mother or the daughter. Therefore, unlike the usual rule in which only the mother centriole could assemble an axoneme, both the parent centrioles in insects are able to nucleate functional sperm axonemes. This suggests that the parent centrioles lost their identity during insect male meiosis or that the daughter centrioles acquire the ability of their mothers. This possibility is supported by the observation that the specific daughter centriole associated protein centrobin accumulates at both the parents in primary *Drosophila* spermatocytes [79].

**Figure 8:**
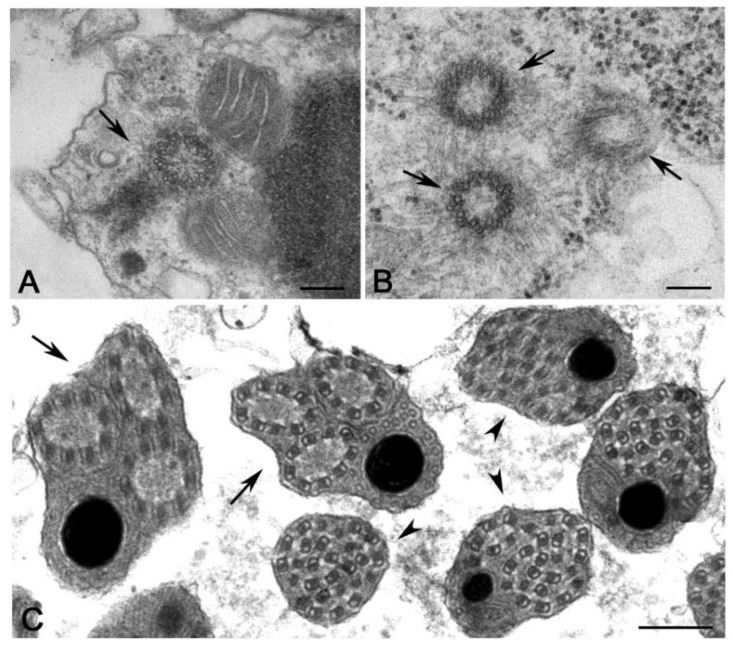
Abnormal male gametogenesis and supernumerary centrioles in differentiating spermatids. Details (**A**) of spermatids of the termite *Reticulitermes* with a pair of orthogonal centrioles (arrow) and (**B**) spermatids of the thrips *Haplothrips* showing three parallel centrioles (arrows) (**B**). (**C**) Each centriole in thrips spermatid organizes distinct axonemes (arrows) that fuse in a single large microtubule bundle (arrowheads). Bars 200 nm.

Exceptions to the usual inheritance of a single centriole during male gametogenesis have been reported in several insect species, principally within the groups characterized by an aberrant meiotic process [160]. In Hymenopteran with haploid males, the conventional meiosis is replaced by a “simple” mitosis giving rise to two haploid spermatids. In this process, as is the rule during mitosis of most animal cell lines, a centriole duplication occurs, and the spermatids inherit a centriole pair. Accordingly, the arrhenotokous parthenogenetic parasitoid *Gryon* lacks male meiosis and each spermatocyte gives rise to two spermatids each containing a couple of orthogonally oriented centrioles [161]. The spermatids of the isopterans *Reticulitermes* (Figure 8A) and *Zootermopsis* [162,163], and the thricopteran Hydroptilidae [164], also contain two orthogonal centrioles. The spermatids of Mallophaga and Anoplura [165], the related Psocoptera [166] and the gall-midge *Semudobia* [167] show two parallel centrioles.

**Figure 9:**
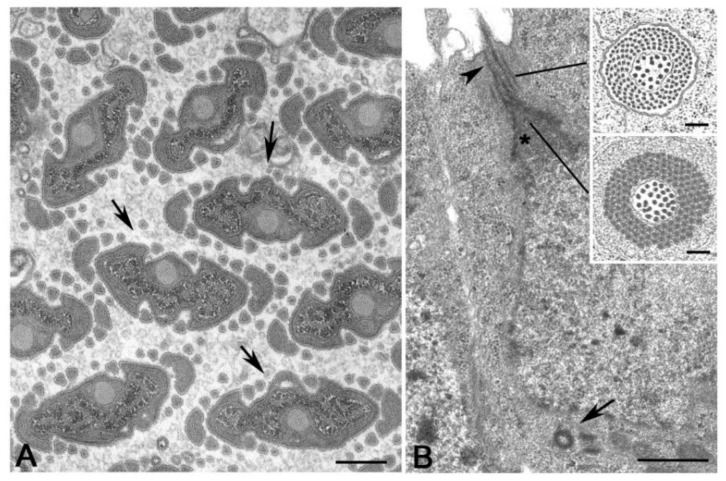
Aflagellate sperm. (**A**) Cross section of aflagellate sperm (arrows) in the homopteran aleyrodid *Bemisia*. (**B**) Spermatid of the archaeococcidae *Matsucoccus* showing a pair of orthogonal centrioles (arrow) and a peripheral bundle of microtubules (arrowhead) emerging from a cluster of dense material (asterisk); insets are cross sections at different levels within the microtubule bundle. Bars: A,B, 1 mm; insets, 200 nm.

Despite the presence of a centriole pair, the spermatids of *Gryon* assemble only one axoneme that is nucleated by the distal centriole, whereas the proximal one has reduced size [161]. Both the parallel centrioles of Mallophaga and Anoplura organize functional axonemes within the same cytoplasm and the sperm is biflagellate [165]. In thrips, that also lack a meiotic process, one of the two centrioles of the spermatids gives origin to a third centriole (Figure 8B). Each centriole nucleates an axoneme that projects outside the cell as a flagellum (Figure 8C). Later, during spermiogenesis, the three flagella fuse their plasma membrane to form a large microtubular bundle of 27 units consisting of doublet and singlet microtubules (Figure 8C) [168,169]. Remarkably, the spermatid of the termite *Mastotermes* has one hundred centrioles that nucleate as many independent flagella [126,127]. By contrast, the centrioles of the spermatids of Hodotermitidae do not organize a structured axoneme and disappear during spermatid differentiation. So, the mature sperm is aflagellate [163]. Centrioles that do not organize axonemes have been also reported in differentiating spermatids of Thricopteran Hydropsychidae [170]. However, some scattered microtubules extend from the distal end of the centrioles in this species but do not organize a true axoneme and soon disappear. Aflagellate sperm cells resulting from the degeneration of the axoneme during spermatid differentiation have been described in several insect species (Figure 9A) [58]. The spermatids of the archaeococcid *Matsucoccus* inherit at the end of spermatogenesis two orthogonal centrioles (Figure 9B). However, these centrioles do not organize a functional axoneme at the onset of spermiogenesis. An axial structure consisting of single microtubules arranged in circular arrays assemble from a peripheral cluster of electron-dense material (Figure 9B) containing g-tubulin [171]. These findings raise the question of how several insect species have elaborated controls over centriole/basal body conversion during spermatogenesis and how they have inactivated the usual switch between centrosome organization and axoneme nucleation.

The spermatogenesis in *Drosophila* seems to exactly mirror the general rule of the insect meiosis that results in the formation of haploid spermatids carrying only one centriole [53]. However, the recent observations of a small procentriole close to the proximal region of the basal body [122,123] open a new scenario on the number of centrioles at the beginning of spermiogenesis in *Drosophila*. Whether such organelle is also present in other insect species is an open question. However, an unstructured cylinder has been reported in the spermatids of the beetle *Tribolium* [159], whereas a distinct procentriole consisting of nine single microtubules has also been observed during the spermiogenesis of the coccinellid beetle *Adalia* [76].

## 8. How Many Centrioles at Fertilization? One, Two or None?

Some quiescent and differentiated cells inactivate or remove their centrioles [172,173,174,175], perhaps to avoid an improper assembly of centrosomes which could lead to unneeded divisions. The female gamete also eliminates or inactivates its centrioles to avoid multiple aster formation at fertilization and failed embryonic development [176,177,178]. Moreover, elimination of the maternal centrioles could eventually prevent spontaneous parthenogenetic development [179]. Therefore, the assembly of the zygotic centrosome at fertilization requires in most animals the direct involvement of the male gamete, which contributes not only its genetic material but also provides its centriole [45,176,180,181]. This is well evident by the findings that the whole sperm enters the oocyte at fertilization in many organisms [181,182,183]. The sperm centriole is not excised from the tail at fertilization and has been observed at one pole of the gonomeric spindle in the early *Drosophila* [184,185,186] and *Chrysopa* [97] embryos. The centriole-axoneme complex persists during the syncytial mitoses in association with the spindle poles of dividing nuclei. Thus, the sperm centriole that is not excised by its axoneme is a true basal body able to duplicate and organize a centrosome, in contrast to the general idea that these functions are incompatible.

During spermiogenesis, most of the centrosomal proteins associated with the centrioles during meiosis disappear and the egg inherits a naked sperm centriole unable to organize a functional centrosome [187]. However, the paternal centriole functions as a magnet that recruits from the egg cytoplasm all the main centrosomal components need for its duplication and microtubule nucleation [176,188]. Interestingly, the reduction during *Drosophila* spermiogenesis of the expression levels of the typical centrosomal proteins Anastral Spindle 1 (Ana1) and Asterless (Asl) [189] is accompanied by the unexpected accumulation of Poc1B [123], a conserved centriolar protein essential for centriole elongation and stability [190].

**Figure 10:**
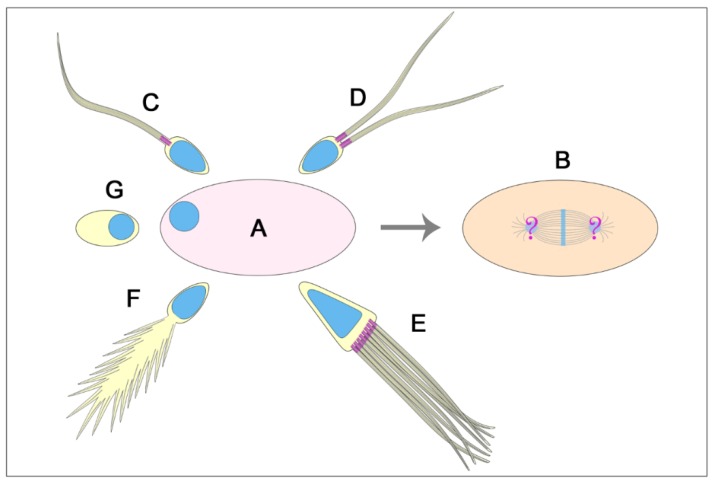
Centriole number in insect spermatids and fertilization problems. The insect egg (**A**) is activated during the transition through the oviduct, but the development in dioic species requires the contribution of the male gamete to assemble the first zygotic spindle (**B**). This process is obvious when the sperm have only one centriole (and eventually an atypical centriole) (most of the insects, (**C**). However, this aspect is less clear when the sperm carry two functional centrioles (Mallophaga and Anoplura, (**D**) or multiple centrioles (*Mastotermes*, (**E**) since in this case the egg is forced to elaborate mechanisms to avoid polyspermy. By contrast, the spermatids with giant axonemes consisting of large cytoplasmic bundles of microtubules, but with modified centrioles (i.e., several gall-midges), (**F**) or aflagellate sperm without centrioles (as in some white flies), (**G**) raise the question of how the egg cytoplasm can assemble the first centrosome without obvious male contribution.

The assembly of the first zygotic spindle, with the exception of mice [191], requires two functional centrosomes each containing a centriole pair. Therefore, the zygotic centrosome consists of two centrioles that duplicate as usually occurs during the mitotic divisions. However, in different animal groups, the sperm provides either one, two or no centrioles at fertilization [176]. It is, therefore, unclear how the first two centrioles are recovered at fertilization. Since the sperm of most animals have a proximal and a distal centriole, they can provide the egg with two centrioles at fertilization [56,192]. However, sperm with a single centriole have been described in several animal groups including insects [56,58]. Therefore, the lack of a second centriole raises the question of how the zygotic spindle is assembled (Figure 10). The most trivial explanation is that the single sperm centriole undergoes two sequential duplication processes to obtain the two centrosomes need to organize the first mitotic spindle. Although, this is a likely possibility, there are not experimental observations to validate this hypothesis. Rather, the procentrioles found in early *Drosophila*, *Adalia* and *Tribolium* spermatids [84,96,159] could represent the precursor of the second centriole need to organize the first zygotic centrosome. However, these procentrioles do not acquire the complete set of microtubules and are no longer found in mature sperm, suggesting that they later disappear. Then, what could be the function of these transient structures? Although we confirm the lack of true procentrioles in mature sperm, we cannot exclude the presence of unstructured centriole precursors able to organize functional centrioles at fertilization. It has been reported that the early *Drosophila* spermatid displays a proximal centriole-like structure (PCL) near the basal end of its centriole [125]. The PCL is composed of typical centriole components and its assembly involves the same proteins required to initiate centriole formation [125,193,194]. Therefore, it is possible that the procentriole acts at the beginning of the spermiogenesis as a recruiting centre for proteins needed to assemble the second centriole at fertilization. The PCL undergoes centrosome reduction during late spermiogenesis but maintains paternal Poc1B that is essential to ensure the proper zygotic centrosome assembly and embryo development [124]. Therefore, the second centriole at fertilization could be provided by the sperm in the form of an atypical centriole. The centriole precursor can recruit pericentriolar material to assemble a functional centrosome and can duplicate to form a new daughter centriole [187]. If it is so, the generally retained inheritance of a single centriole at fertilization in insects must be revised.

There are several examples of insect sperm in which the single centriole is highly modified after the assembly of the axoneme and its typical barrel-shaped organization is no longer visible. In *Sciara* the centriole is fragmented [195] whereas no centriole remnants have been observed in Cecidomyiids with aberrant axonemes [58]. How can the egg of these species inherit functional centrioles? In mammals, the distal centriole loses its organization forming an atypical structure, or atypical centriole, that recruits centrosomal proteins and duplicates while maintaining its attachment to the axoneme [159]. However, the mammalian sperm have a proximal barrel-shaped centriole. It is unclear if the insect sperm with highly modified axonemes have centriole precursors.

Enigmatic is the centriole inheritance when the aflagellate sperm is acentriolar as it occurs in some species of protura [196], isoptera [163], ephemeroptera [197] homoptera [198,199] and diptera [200]. A simple explanation could be that the egg cytoplasm self-organizes the zygotic centriole in these species. But if it is so, why these eggs do not develop parthenogenetically? Egg activation in insects does not require the interaction with the sperm, and the parthenogenetic development usually starts with the formation *ex novo* of the centrioles within the egg cytoplasm [40,201,202]. Alternatively, the acentriolar sperm could provide an unstructured framework able to recruit the maternal components needed to organize the first zygotic centriole [187]. This hypothesis could be supported by the observation that the PCL found in the early *Drosophila* spermatids disappears in mature sperm, but its associated Poc1B is delivered to the egg [124]. Therefore, Poc1B could represent the scaffold to recruit some maternal centrosome components. However, whether the PCL or Poc1B are present in the acentriolar sperm is unknown.

In contrast, the sperm of Mallophaga and Anoplura [165] and Psocoptera [164], that have two parallel centrioles and the unique sperm of *Mastotermes* with one hundred centrioles [127,128] raise important questions about the presence of too many centrioles at fertilization. One problem during fertilization of many organisms is the prevention of the polyspermy. Such process blocks the entrance in the egg cytoplasm of more than one sperm to avoid the formation of supernumerary asters and then the failure of embryo development [203]. So, how, can be reconciled the presence of several centrioles in the fertilizing sperm and the proper embryo development? It has been shown that more than one sperm could be occasionally present in the *Drosophila* egg at fertilization and that the polyspermic eggs vary from 1 to 6% depending on the *Drosophila* species [204,205]. However, the supernumerary sperm do not hinder the development of these eggs. The supernumerary sperm replicate their DNA, duplicate their centrioles that organize a bipolar spindle, but mitosis arrests at the beginning of anaphase [204]. How mitosis of the supernumerary sperm is arrested represents a conundrum. However, such mechanism could ensure the correct zygotic development in the insect species with multiple centrioles in their sperm.

## 9. The Peculiar Tale of the Progressive Loss of Sperm Flagella and the Re-Acquisition of Sperm Motility

Homoptera Sternorrhyncha displays a peculiar evolutive trend leading to the loss of sperm flagella and motility [206]. Aphidoidea have a conventional centriole and basal body producing a sperm with a 9 + 9 × 2+2 typical and motile flagellar axoneme. Psylloidea shows a normal centriole and basal body which gives rise to a posteriorly flattened flagellar axoneme. In Aleyrodoidea, likely the sister group of Coccoidea and Aphidoidea, the centriole is an aberrant structure giving origin to an apparently normal flagellar axoneme with doublets devoid of dynein arms and without central complex. At maturity, the sperm flagellum is no longer visible with the typical structure, but it is longitudinally crossed by a solid rod of tubules and it is immotile [198,199]. Coccoidea, the most specialized Sternorrhyncha, have sperm that have lost a true flagella axoneme. However, the sperm motility is supported by a bundle of singlet microtubules interconnected by short bridges of a dynein–like protein [207]. In the archaeococcid *Matsucoccus,* the early spermatids have a conventional non-functional centriole that does not give origin to the axoneme, but a microtubule-dependent structure is organized far from the centrioles by a peripheral non-centrosome organizing center [171]. This finding supports the hypothesis about the “autonomous” centriole origin through a sequence of events starting from a bundle of polarized microtubules that later should have contacted the plasma membrane and then protruded outward the cell to give origin to an atypical motile structure [208,209,210,211]. A centriole could have taken origin at the proximal end of the polarized microtubule bundle.

## 10. Concluding Remarks

Hexapoda is the largest subphylum within the phylum Arthropoda, and among them over a million of the Insect species have been described from terrestrial and aquatic habitats, thus representing the most successful group of organisms on Earth. Therefore, Hexapoda could be a living lab enabling investigations into the structure, the assembly, the dynamic changes and the function of the centrioles during the cell life in a broad range of tissues and different developmental contexts.

The variety of the Hexapoda often reflects unexpected variations of the centriole architecture and/or the ciliary structures that they organize. One interesting finding is the presence of centrioles with doublet microtubules in lower Hexapoda, whereas the typical insect centriole usually consists of triplets, confirming the evolutionary separation between the smaller wingless orders Collembola and Protura and the class Insecta. However, centrioles with triplet microtubules firstly appear in Diplura that are the third order of non-insect Hexapoda supporting the placement of this order as sister-group of the Insects [95]. The acquisition of the C-tubule, mandatory for the formation of the accessory tubules in the sperm axoneme, is restricted to germ cell centrioles, whereas somatic centrioles maintain the doublet microtubules. Moreover, centrioles with unusual structure diverging from the common nine-fold symmetry are found within unrelated Hexapoda groups. This finding brought into the question the role of the cartwheel and of the related Sas6 protein in centriole assembly and duplication, as reported in conventional model organisms. Finally, there are many examples among the Hexapoda of centrioles able to recruit centrosomal material during male gametogenesis but unable to organize functional axonemes. This aspect raises important questions on the temporal control of the centriole function. Thus, a better understanding of the centriole biogenesis in various animal groups would allow us to gain insight in the general mechanisms of centriole formation and function.
